# Acute Effects of a Single Whole-Body Vibration Session on Mobility and Postural Control in Community-Dwelling Older Adults: A Randomized Clinical Trial

**DOI:** 10.3390/jfmk10010075

**Published:** 2025-02-24

**Authors:** Gustavo Christofoletti, Azriel Cancian Nepomuceno de Almeida, Camilly Lorentz, Sidney Afonso Sobrinho, Renata Terra de Oliveira, Suzi Rosa Miziara Barbosa

**Affiliations:** Institute of Health, School of Medicine, Federal University of Mato Grosso do Sul, UFMS, Campo Grande 79060-900, Brazil

**Keywords:** exercise, aged, postural balance, gait analysis, physical therapy modalities

## Abstract

**Background:** Whole-body vibration is a modality of exercise that uses high-frequency mechanical stimuli to enhance motor functions. Previous studies have demonstrated benefits of whole-body vibration in older adults. However, prolonged use of this modality of exercise may be detrimental to certain conditions. **Objectives**: to verify the acute effects of a single whole-body vibration session on mobility and postural control in community-dwelling older adults. **Methods**: In this two-arm, single-blind clinical trial, fifty-two participants were randomly allocated to either the experimental (subject to a single whole-body vibration session with a vibration amplitude of 2 mm and a frequency of 40 Hz) or placebo group. The exercise sessions were conducted using a tri-planar vibration platform. The tri-plane plates were adjusted to vibrate up and down, side to side, and front to back. The assessments included mobility and postural control tests. Repeated-measures analyses of variance were performed to examine the main effect of group (experimental vs. placebo), time (baseline vs. after the intervention), and group × time interaction effect. Significance was set at 5%. **Results**: Compared with the placebo group, participants who underwent whole-body vibration showed positive outcomes in terms of mobility (*p* = 0.014, effect size: 0.115). Contrastingly, no significant differences were observed between the groups in terms of postural control (*p* > 0.05). **Conclusions**: Benefits of a single whole-body vibration session were observed on mobility. Using whole-body vibration to improve postural control may require additional sessions. Contraindications typical of aging should be taken into account.

## 1. Introduction

The global population is aging, driven by improved life expectancy and quality of life. Simultaneously, families have adopted birth control measures that have contributed to a decline in the number of births each year. As life expectancy increases and birth rates decrease, the proportion of older people has been elevated. This demographic shift raises concerns about maintaining health while ensuring quality of life and functional capacity in older adults [1,2,3].

Aging involves gradual changes in all systems of the human body. This process results in increased susceptibility to diseases and a decline in functional capacities [4,5,6]. Compared with younger people, older adults are more likely to experience comorbidities and limitation. This situation imposes a significant burden on older adults and families, with the increased use of medications, medical procedures, and hospital services [7,8]. As a consequence, there is a need to explore alternative models, resources, and therapies aimed at preventing and controlling age-related diseases.

Physical therapists and physical education professionals play an important role in promoting the health of older adults. Exercise is a preventive and therapeutic strategy for individuals of all ages, offering benefits in terms of strength, balance, coordination, and flexibility. Specifically, exercises are important for minimizing the effects of geriatric syndromes, such as frailty, falls, depression, and cognitive decline [9,10]. The benefits of exercise are substantial for older adults, which led the World Health Organization to recommend a weekly dose of exercise for all individuals. Unfortunately, in many cases, this is not what happens [11].

A wide range of exercises offer significant benefits in older adults. Mind-body practices such as Pilates, Yoga, and Tai Chi Chuan are important for improving both physical and mental health [12,13,14]. Aerobic, resistance, and dual-task exercises are effective alternatives for preventing falls [15,16]. Cognitive-motor activities are important for the activation of specific brain areas, such as memory, attention, and executive functions [17]. Isolated exercises, such as whole-body vibration (WBV), have shown benefits in older individuals by activating motor neurons [18,19].

WBV is a modality of exercise that uses high-frequency mechanical stimuli generated by a vibrating platform transmitted through the body. Commonly used in sports, fitness training, and rehabilitation settings, the WBV therapy involves the participant standing on the platform while trained professionals help adjust the duration, amplitude, and intensity to suit individual needs. WBV requires isometric contraction to maintain a slight knee flexion during the session. Its benefits have been linked to sinusoidal vibrations, which deliver mechanical stimuli that trigger rapid co-contractions of muscles [19,20].

Previous studies suggest that WBV may offer benefits for older adults. For instance, Pillay et al. [21] and Sañudo et al. [22] reported that specific WBV protocols reduced the risk of falls and enhanced strength, balance, mobility, gait, and overall physical performance. Gonçalves de Oliveira et al. [23], on the other hand, found no significant effects of WBV on muscle endurance and lower limb power.

Although WBV has shown benefits, some studies have raised concerns about its prolonged use. For example, Cardinale and Popel [24] identified low back pain as a potential side effect of WBV. Pasqualini et al. [25], in a study on mature male rats, reported deleterious effects on bone density, including increased cortical thickness in the femur diaphysis along with decreased porosity. Additionally, Monteleone [26] identified WBV as a possible contraindication for individuals with kidney stones.

Considering that safer WBV protocols involve low mechanical impedance [27], this study evaluated the acute effects of a single 5 min WBV session on mobility and postural control in community-dwelling older individuals. The authors hypothesized that, despite its short duration, a single WBV session could lead to improvements in mobility and postural control in older individuals.

## 2. Materials and Methods

This was a single-blinded, randomized clinical trial with two parallel groups. This study was conducted in the Outpatient Clinic of the Federal University of Mato Grosso do Sul, Campo Grande, Brazil. The study was conducted in accordance with the CONSORT statement, the Declaration of Helsinki, and the guidelines for good clinical practice. All participants signed a consent form prior to the assessment. The protocol was approved by the institution’s ethics committee (# 2.355.458) and prospectively registered in the Brazilian Registry of Clinical Trials (# RBR-2mw2kwv).

The sample consisted of 52 individuals, 25 women and 27 men, with a mean age of 67.7 ± 6.4 years. All participants were randomly allocated to either the experimental (intervention) or the placebo (no intervention) group. Individuals were eligible for inclusion if they were 60 years or older; of both sexes; of any race or creed; sedentary; and had no neurological, psychiatric, or musculoskeletal problems. The exclusion criteria were participants living in long-term care institutions, those who had undergone recent surgery (<6 months), and those unable to attend to the outpatient clinic.

### 2.1. Sample Size

The sample size was estimated using the G*Power software version 3.1.9.7 (Heinrich Heine Universität Düsseldorf, Düsseldorf, Germany). The parameters included in the sample size analysis were based on the effect sizes reported by Fischer et al. [28], which ranged from 0.18 (for mobility) to 0.28 (for gait). We standardized a conservative effect size of 0.20, with a power of 0.8 and a significance level of 0.05. The analysis indicated that 52 participants were required to control for Type I and Type II statistical errors.

### 2.2. Selection, Randomization, and Monitoring

Participants were recruited directly by the researchers and through social media platforms. Researchers screened potentially eligible participants from those who expressed interest in the study. Of the 60 older individuals initially interested, eight were unable to attend the outpatient clinic. After participant recruitment, an independent researcher performed randomization to assign participants to either the experimental or placebo group.

**Figure 1:**
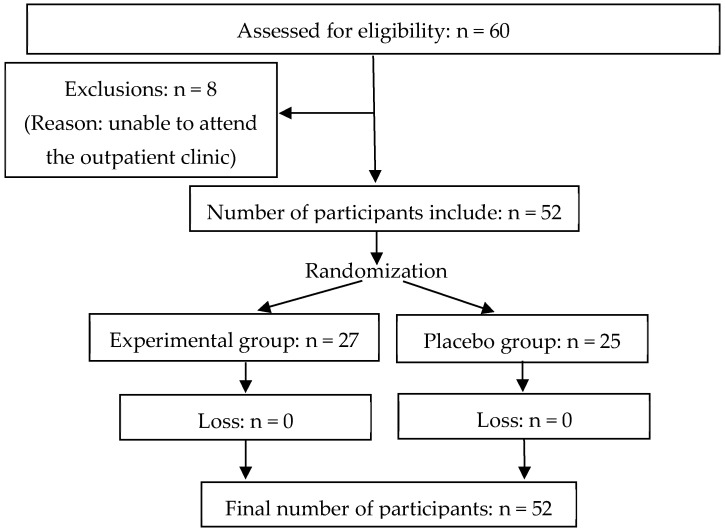
Flow diagram of the study.

The division of the groups was based on the stratified randomization method to ensure a balanced and comparable distribution of participants. Each participant selected an opaque envelope containing either the label “intervention group” or “placebo group”, determining whether they received treatment on the vibration platform or underwent a placebo intervention. Figure 1 details the flow of participant selection and monitoring.

### 2.3. Blinding

**Figure 2:**
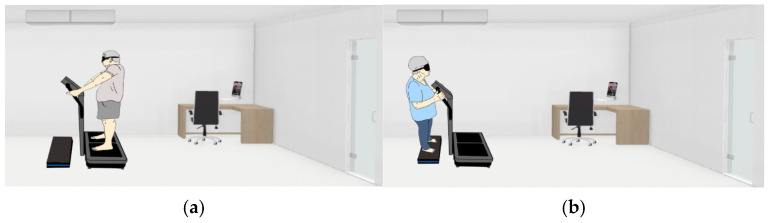
Specific model demonstrating the experimental (**a**) and the placebo (**b**) exercises.

In this study, all participants were evaluated by a single evaluator. The evaluator was a physical therapist specialized in movement disorders and who had experience with the equipment used in the study. Since the authors evaluated all participants in the same location used for the intervention (to avoid any potential effect of sensory stimuli other than WBV), blinding the assessor was not feasible. The participants were blinded to the intervention. All the participants wore blindfolds during the test and could not see the group to which they were assigned (experimental or placebo). A step was positioned in front of the vibration platform to create a placebo effect in the no-intervention group. The platform was switched on in both groups. Participants in the placebo group placed their hands on the vibration platform, creating an illusion of experiencing vibration. Figure 2 presents a model illustrating participants performing both the experimental and placebo exercises.

### 2.4. Therapeutic Protocol

The sessions were conducted using a tri-planar vibration platform. The tri-plane plates were adjusted to vibrate up and down, side to side, and front to back. The platform measured 80 cm in length, 80 cm in width, and 170 cm in height. The participants stood barefoot and blindfolded throughout the test.

The experimental group participated in a 5 min WBV protocol with a vibration amplitude of 2 mm across all planes of motion and a frequency of 40 Hz. These parameters were selected by the authors because they are considered safe and unlikely to pose any risks to the participants [27]. The participants in the placebo group stood barefoot and blindfolded on a step that matched the length (80 cm) and width (80 cm) of the vibration platform. The step was positioned in front of a vibration platform (Figure 2). The vibration platform was kept on for 5 min to create an illusion to the participants that they were on the platform. The participants in both groups were instructed to step onto the device (vibration platform or step) with both feet, remain standing, and hold onto the handrails. The researchers asked the participants to keep their knees and hips slightly flexed during the task.

### 2.5. Evaluative Assessment

All the participants were screened in the same evaluation room where the vibration platform was located. This design aimed to evaluate the acute effects of WBV while minimizing any sensory influence from moving participants to another room. The main outcome measures were mobility and postural control, assessed at baseline and re-evaluated immediately after the intervention. The order of the tests was randomized to prevent any potential bias.

Mobility was assessed using the Timed Up and Go (TUG) test [29]. This test measures the time required for a person to rise from an armchair, walk a distance of 3 m, turn, walk back to the chair, and sit down. A longer time to complete the task indicates a higher risk of falls. Postural control was assessed using the BIOMEC 400_V4 (EMG System^®^, São José dos Campos, Brazil) and Baro Scan (HS Technology^®^, Londrina, Brazil) platforms.

The BIOMEC 400_V4 is a force platform featuring a 2500 cm^2^ plate (50 cm × 50 cm) equipped with four load cells and a 100 Hz calibration system. Participants performed all tests barefoot and were instructed to stand on the platform for 60 s with their feet positioned 10 cm apart. The platform measured three moments of force (Fx, Fy, and Fz) while participants were standing. Data were processed using MATLAB^®^ (The MathWorks, Natick, MA, USA). The assessed variables included frontal and lateral sway (defined as any movement of the top of a vertical member relative to its base, measured in cm), center of pressure (calculated as an elliptical base in cm^2^, representing its change over time), and frontal and lateral speed (defined as the speed deviation of the center of pressure, measured in cm/s). Greater sway in the frontal and lateral directions, along with a larger center of pressure sway area and faster imbalance speed, indicate poorer postural balance.

The Baro Scan platform is a digital baropodometric system composed of 4096 sensors that capture high-definition images of foot pressure. It measures weight bearing on the right and left sides of the body, as well as maximum pressure on the right and left sides. The participants performed all tests barefoot. Discrepancies in weight bearing and pressure between the feet are indicative of poorer postural control.

### 2.6. Statistical Analysis

Statistical analysis was performed in several steps. First, the data were characterized using means and standard deviations. Second, the parametric assumptions of the data were checked for all variables (normality tests, homogeneity of variance tests, and expected vs. observed Q-Q plots). For data that did not meet the assumptions of normality and homogeneity of variance, logarithmic transformation was applied. Independent Student’s *t*-tests and chi-squared tests were used to analyze the anthropometric characteristics of the groups. Finally, repeated-measures analysis of variance (ANOVA) was performed to examine the main effects of group (experimental vs. placebo), time (baseline vs. after the intervention), and the group × time interaction effect. Partial eta squared analyses were used to accurately assess the effect size (ES) of the intervention. ES was reported when significance was achieved (*p* < 0.05).

## 3. Results

In total, 60 participants were recruited. Eight individuals were excluded, citing difficulties in attending the outpatient clinic. Fifty-two participants completed the study (27 in the experimental group and 25 in the placebo group). No participants dropped out during the interventions. Table 1 shows the characteristics of the groups in terms of sample size, age, height, weight, and body mass index.

### 3.1. Mobility

Repeated-measures analysis of variance revealed that WBV had a beneficial effect on the TUG test. In other words, participants in the experimental group performed the test faster after the WBV intervention than those in the placebo group. Table 2 shows the initial and final mobility scores for each group on the TUG test.

### 3.2. Postural Control

Table 3 shows the initial and final scores of the participants on postural control. No significant group × time interactions were observed when comparing the experimental and placebo groups. However, a significant effect of time was found for lateral speed, and a significant group effect was observed for maximum pressure on the left side.

None of the participants abandoned either the experimental or placebo protocols. The participants in the experimental group reported no adverse effects and tolerated the WBV section. Specifically, none of the participants experienced fatigue, nervousness, headache, vertigo, difficulty concentrating, or mood changes.

## 4. Discussion

This study aimed to evaluate the acute effects of a single WBV session on mobility and postural control in community-dwelling older adults. The results confirmed the hypothesis that individuals who underwent a unique WBV session experienced improvements on mobility. However, no differences were observed in postural control. These findings are relevant to clinical practice.

Fifty-two older adults with no neurological or musculoskeletal disorders completed this study. This group of participants was chosen because of the importance of maintaining functional capacity and preventing geriatric syndromes. The participants were randomly allocated, and external factors that could affect the outcomes, such as age, physical activity level, and anthropometric data, were rigorously controlled. As shown in Table 1, the groups were homogeneous across all the variables. This is important as it ensured that such variables did not introduce any bias into the findings.

To date, most studies assessing the benefits of a single WBV session have been conducted in young adults. Maslova et al. [30] assessed 70 young adults and found that WBV offers acute benefits in enhancing proprioceptive response. Given that in this study postural control did not show any significant benefit, the results suggest that a single WBV session may not provide the same proprioceptive advantages for older adults as it does for younger individuals. This difference could be attributed to problems in the proprioceptive system, which is known to be affected by aging [31,32,33]. As WBV targets proprioceptive receptors, it is likely that multiple sessions are necessary to activate receptors effectively in older adults. Further studies are needed to confirm this hypothesis.

Walking ability is important for maintaining functional independence in all age groups. In older adults, several factors can negatively affect walking [33,34,35]. In this study, WBV improved the time taken to complete the TUG test. Participants in the experimental group completed the TUG test more quickly after WBV, whereas those in the placebo group performed more slowly.

Although the difference on the TUG test was statistically significant, it may not be clinically relevant [36]. As shown in Table 2, WBV resulted in a 0.5 s improvement on the TUG test, which is noteworthy but may not have significant clinical implications. The results suggest that a single session of WBV improves mobility, but additional sessions may be important for improving performance more effectively. Combining WBV with other therapies, such as conventional physical therapy, may yield better outcomes in older adults. Previous studies show promising results from combining WBV with exercise for older individuals, both with and without neurological conditions [37,38].

Pre- and post-session comparisons showed no benefit from WBV on postural control. Table 3 shows group differences for maximum pressure on the left side and time differences for lateral speed. However, no postural control variable showed a significant “group × time interaction”, which would indicate a specific benefit from WBV.

The authors attribute the lack of improvement in postural control with WBV to three main factors. First, enhancing postural control in a single session may be challenging, as several complex systems influence static balance in older individuals [39]. Although a single session resulted in improvements in mobility, as previously detailed, the statistical significance of these findings does not necessarily translate to clinical relevance. Second, improving postural control in individuals without previous neurological or sensory conditions (as in this sample) may be more challenging than in older adults with a history of neurological or sensory conditions. As an example, Yoosefinejad et al. [40] identified positive effects of a single WBV session in individuals with neuropathy. Third, the frequency of the vibration platform may have influenced the outcomes. Although Mahbub et al. [41] found that WBV did not improve postural control at frequencies of 15, 20, or 25 Hz, our frequency of 40 Hz also showed no effect on postural control. Therefore, further studies are needed to explore the acute effects of WBV in older adults using different parameters of the vibration platform.

There is no consensus on the ideal frequency for vibration platforms. Using the formula *a*_peak_ = (2*πf*)^2^⋅*A*, where *a*_peak_ is the maximum acceleration experienced by an object during a vibration, *f* is the frequency in Hz, and *A* is the amplitude in meters, a frequency of 40 Hz with an amplitude of 2 mm could generate a peak acceleration of approximately 12.9 g (*a*_peak_ = (2*π* × 40)^2^ × 0.002 ≈ 12.9 g). On one hand, this may be uncomfortable for some participants and, in certain cases, could exceed safety thresholds for prolonged exposure. However, on the other hand, since this was a short, single-session protocol, no participants reported any discomfort or side effects.

The findings of this study should be interpreted with caution considering some important limitations. First, the results are limited to older adults with no prior neurological, psychiatric, or musculoskeletal disorders. Second, the findings are specific to a frequency of 40 Hz for a unique 5 min session. Third, we did not examine the long-term retention of WBV benefits on mobility. Future studies should explore different parameters of the vibrating platform within this population and assess whether WBV-related improvements on mobility can be maintained. Fourth, although none of the participants in the experimental group reported any fatigue symptoms, it is possible that a 5 min session may have caused a small level of fatigue that was not controlled in this study.

It is important to recognize that, as the placebo group was exposed to upper limb vibration with their hands on the vibration platform (to ensure complete blinding of that group), it is possible that some vibration effect was observed in this group. Xu et al. [42] identified that vibration exercises on upper limbs are effective in enhancing strength and power performance. However, unlike Xu et al. [42], who provided an 8-week intervention, the authors believe it is unlikely that 5 min of upper limb shaking could have provided significant benefits to the placebo group. To confirm this hypothesis, the authors recommend further studies with a non-shaking placebo group, which would yield important findings, although it may not allow for complete blinding of that group.

## 5. Conclusions

A single session of WBV had a positive effect on mobility in community-dwelling older adults. Conversely, the unique WBV session did not show any benefit on postural control. Despite the limitations of this study, the results support the use of WBV in older adults. Further studies are necessary to investigate whether additional sessions of WBV would improve postural control in older individuals and whether combining techniques, such as WBV and conventional physical therapy, may yield better results.

## Figures and Tables

**Table 1:** General characteristics of the groups.

Variables	Groups		95% C.I of the Difference	*p*
	Experimental (n = 27)	Placebo (n = 25)		
Sex (male:female), n	15:12	12:13	0.7; 0.8	0.586
Age, years	68.4 (7.2)	67.1 (5.6)	−2.3; 4.9	0.466
Height, m	1.7 (0.1)	1.6 (0.1)	−0.1; 0.1	0.120
Weight, kg	75.6 (14.1)	70.1 (11.3)	−1.6; 12.7	0.127
Body mass index, kg/m^2^	27.5 (3.8)	27.1 (3.5)	−1.6; 2.5	0.688

Note: Chi-square tests were used for categorical variables (sex) and independent Student’s *t*-tests were used for the continuous variables.

**Table 2:** Initial and final assessment of the participants’ mobility.

Variables	Groups	Assessments		ANOVA Main Effect		
		Initial	Final	Group	Time	Interaction
TUG, s	Experimental	7.8 (1.3)	7.4 (0.9)	*p* = 0.301	*p* = 0.892	*p* = 0.014
	Placebo	7.8 (1.4)	8.2 (1.5)			ES: 0.115

Note: Repeated-measures analysis of variance was used to examine the main effect of group (experimental vs. placebo), time (baseline vs. after the intervention), and group × time interaction effect.

**Table 3:** Initial and final assessment of the participants on postural control.

Variables	Groups	Assessments		ANOVA Main Effect		
		Initial	Final	Group	Time	Interaction
Frontal sway, cm	Experimental	3.6 (1.8)	3.4 (1.4)	*p* = 0.331	*p* = 0.725	*p* = 0.304
	Placebo	3.1 (1.1)	3.2 (1.4)			
Lateral sway, cm	Experimental	2.1 (1.1)	2.1 (0.9)	*p* = 0.487	*p* = 0.320	*p* = 0.910
	Placebo	2.0 (1.3)	2.1 (1.4)			
Center of pressure, cm^2^	Experimental	3.7 (5.0)	3.8 (3.4)	*p* = 0.338	*p* = 0.260	*p* = 0.893
	Placebo	3.4 (5.4)	3.6 (5.5)			
Frontal speed, cm/s	Experimental	1.7 (0.6)	1.6 (0.6)	*p* = 0.875	*p* = 0.789	*p* = 0.188
	Placebo	1.6 (0.6)	1.7 (0.5)			
Lateral speed, cm/s	Experimental	1.2 (0.4)	1.1 (0.3)	*p* = 0.462	*p* = 0.024	*p* = 0.994
	Placebo	1.3 (0.4)	1.2 (0.2)		ES: 0.098	
Weight bearing on the right side of the body, %	Experimental	46.4 (4.5)	47.1 (5.1)	*p* = 0.184	*p* = 0.355	*p* = 0.860
	Placebo	48.3 (6.0)	48.4 (5.9)			
Weight bearing on the left side of the body, %	Experimental	53.1 (4.7)	52.8 (5.1)	*p* = 0.135	*p* = 0.871	*p* = 0.775
	Placebo	51.0 (5.0)	51.1 (5.9)			
Maximum pressure on the right side, Kgf	Experimental	1.2 (0.4)	1.2 (0.4)	*p* = 0.413	*p* = 0.850	*p* = 0.656
	Placebo	1.1 (0.4)	1.1 (0.5)			
Maximum pressure on the left side, Kgf	Experimental	1.4 (0.7)	1.4 (0.7)	*p* = 0.028	*p* = 0.301	*p* = 0.814
	Placebo	1.1 (0.3)	1.0 (0.4)	ES: 0.092		

Note: Repeated-measures analysis of variance was used to examine the main effect of group (experimental vs. placebo), time (baseline vs. after the intervention), and group × time interaction effect.

## Data Availability

The original contributions presented in the study are included in the article; further inquiries can be directed to the corresponding author.

## References

[1] Beard J.R., Officer A., de Carvalho I.A., Sadana R., Pot A.M., Michel J.P., Lloyd-Sherlock P., Epping-Jordan J.E., Peeters G.M.E.E.G., Mahanani W.R. (2016). The World report on ageing and health: A policy framework for healthy ageing. Lancet.

[2] Partridge L., Deelen J., Eline Slagboom P.E. (2018). Facing up to the global challenges of ageing. Nature.

[3] Leirós-Rodrígues R., Romo-Pérez V., García-Soidán J.L., Soto-Rodríguez A. (2018). Prevalence and factors associated with functional limitations during aging in a representative sample of Spanish population. Phys. Occup. Ther. Geriatr..

[4] Gasmi A., Chirumbolo S., Peana M., Mujawdiya P.K., Dadar M., Menzel A., Bjørklund G. (2021). Biomarkers of Senescence during Aging as Possible Warnings to Use Preventive Measures. Curr. Med. Chem..

[5] Behfar Q., Ramirez Zuniga A., Martino-Adami P.V. (2022). Aging, Senescence, and Dementia. J. Prev. Alzheimers Dis..

[6] Mishra D., Mohapatra L., Tripathi A.S., Paswan S.K. (2024). The influential responsibility of sirtuins in senescence and associated diseases: A review. J. Biochem. Mol. Toxicol..

[7] Majumdar U.B., Hunt C., Doupe P., Baum A.J., Heller D.J., Levine E.L., Kumar R., Futterman R., Hajat C., Kishore S.P. (2019). Multiple chronic conditions at a major urban health system: A retrospective cross-sectional analysis of frequencies, costs and comorbidity patterns. BMJ Open.

[8] Chen L.K. (2021). Towards Appropriate Prescribing for Older Persons with Multiple Chronic Conditions. Arch. Gerontol. Geriatr..

[9] Chodzko-Zajko W.J., Proctor D.N., Fiatarone Singh M.A., Minson C.T., Nigg C.R., Salem G.J., Skinner J.S., American College of Sports Medicine (2009). American College of Sports Medicine position stand. Exercise and physical activity for older adults. Med. Sci. Sports Exerc..

[10] Terra de Oliveira R., Lino T.B., Scarmagnan G.S., Miziara Barbosa S.R., de Souza Pegorare A.B.G., Christofoletti G. (2024). A controlled clinical trial on the effects of aquatic exercise on cognitive functions in community-dwelling older adults. Brain Sci..

[11] Boente-Antela B., Leirós-Rodríguez R., García-Soindán J.L. (2022). Compliance with the recommendations of the World Health Organization on the practice of physical activity in people over 65 years in Spain. J. Hum. Sport Exerc..

[12] Martens N.L. (2022). Yoga Interventions Involving Older Adults: Integrative Review. J. Gerontol. Nurs..

[13] Fernández-Rodríguez R., Álvarez-Bueno C., Cavero-Redondo I., Torres-Costoso A., Pozuelo-Carrascosa D.P., Reina-Gutiérrez S., Pascual-Morena C., Martínez-Vizcaíno V. (2022). Best Exercise Options for Reducing Pain and Disability in Adults with Chronic Low Back Pain: Pilates, Strength, Core-Based, and Mind-Body. A Network Meta-analysis. J. Orthop. Sports Phys. Ther..

[14] Chen Y., Qin J., Tao L., Liu Z., Huang J., Liu W., Xu Y., Tang Q., Liu Y., Chen Z. (2023). Effects of Tai Chi Chuan on Cognitive Function in Adults 60 Years or Older with Type 2 Diabetes and Mild Cognitive Impairment in China: A Randomized Clinical Trial. JAMA Netw. Open.

[15] Scarmagnan G.S., Lino T.B., Pimentel D.E., Silva A.V.B., da Silva Ramos I.M., Christofoletti G. (2024). Benefits of a Dual-Task Training on Motor and Cognitive Functions in Community-Dwelling Older Adults: A Controlled Clinical Trial. Am. J. Phys. Med. Rehabil..

[16] Sherrington C., Fairhall N.J., Wallbank G.K., Tiedemann A., Michaleff Z.A., Howard K., Clemson L., Hopewell S., Lamb S.E. (2019). Exercise for preventing falls in older people living in the community. Cochrane Database Syst. Rev..

[17] Zhang M., Jia J., Yang Y., Zhang L., Wang X. (2023). Effects of exercise interventions on cognitive functions in healthy populations: A systematic review and meta-analysis. Ageing Res. Rev..

[18] Tan X., Jiang G., Zhang L., Wang D., Wu X. (2023). Effects of Whole-Body Vibration Training on Lower Limb Muscle Strength and Physical Performance Among Older Adults: A Systematic Review and Meta-analysis. Arch. Phys. Med. Rehabil..

[19] Yang F., King G.A., Dillon L., Su X. (2015). Controlled whole-body vibration training reduces risk of falls among community-dwelling older adults. J. Biomech..

[20] Wadsworth D., Turnbull J., Lark S. (2022). Psychological Effects of whole-body vibration training in frail older adults: An open, randomized control trial. J. Aging Phys. Act..

[21] Pillay J., Gaudet L.A., Saba S., Vandermeer B., Ashiq A.R., Wingert A., Hartling L. (2024). Falls prevention interventions for community-dwelling older adults: Systematic review and meta-analysis of benefits, harms, and patient values and preferences. Syst. Rev..

[22] Sañudo B., Reverte-Pagola G., Seixas A., Masud T. (2024). Whole-Body Vibration to improve physical function parameters in nursing home residents older than 80 years: A systematic review with meta-analysis. Phys. Ther..

[23] Gonçalves de Oliveira R., Coutinho H.M.E.L., Martins M.N.M., Bernardo-Filho M., de Sá-Caputo D.D.C., Campos de Oliveira L., Taiar R. (2023). Impacts of whole-body vibration on muscle strength, power, and endurance in older adults: A systematic review and meta-analysis. J. Clin. Med..

[24] Cardianel M., Pope M.H. (2003). The effects of whole-body vibration on humans: Dangerous or advantageous?. Acta Physiol. Hung..

[25] Pasqualini M., Lavet C., Elbadaoui M., Vanden-Bossche A., Laroche N., Gnyubkin V., Vico L. (2013). Skeletal site-specific effects of whole body vibration in mature rats: From deleterious to beneficial frequency-dependent effects. Bone.

[26] Monteleone G., De Lorenzo A., Sgroi M., De Angelis S., Di Renzo L. (2007). Contraindications for whole body vibration training: A case of nephrolithiasis. J. Sports Med. Phys. Fit..

[27] Abercromby A.F., Amonette W.E., Layne C.S., McFarlin B.K., Hinman M.R., Paloski W.H. (2007). Vibration exposure and biodynamic responses during whole-body vibration training. Med. Sci. Sports Exerc..

[28] Fischer M., Vialleron T., Laffaye G., Fourcade P., Hussein T., Chèze L., Deleu P.A., Honeine J.L., Yiou E., Delafontaine A. (2019). Long-Term Effects of Whole-Body Vibration on Human Gait: A Systematic Review and Meta-Analysis. Front. Neurol..

[29] Podsiadlo D., Richardson S. (1991). The timed “Up & Go”: A test of basic functional mobility for frail elderly persons. J. Am. Geriatr. Soc..

[30] Maslova O., Shusharina N., Videnin A., Pyatin V. (2024). Integrative function of proprioceptive system in the acute effects of whole body vibration on the movement performance in young adults. Front. Sports Act. Living.

[31] Cressman E.K., Salomonczyk D., Henriques D.Y. (2010). Visuomotor adaptation and proprioceptive recalibration in older adults. Exp. Brain Res..

[32] Toosizadeh N., Ehsani H., Miramonte M., Mohler J. (2018). Proprioceptive impairments in high fall risk older adults: The effect of mechanical calf vibration on postural balance. Biomed. Eng. Online.

[33] Franz J.R., Shelton A.D., Takahashi K.Z., Allen J.L. (2025). Plantar sensation associates with gait instability in older adults. J. Neuroeng. Rehabil..

[34] Kyrdalen I.L., Thingstad P., Sandvik L., Ormstad H. (2019). Associations between gait speed and well-known fall risk factors among community-dwelling older adults. Physiother. Res. Int..

[35] Ichihashi N., Ikezoe T., Sato S., Ibuki S. (2019). Gait asymmetry assessment for older adults by measuring circular gait speed. Geriatr. Gerontol. Int..

[36] Alfonso-Rosa R.M., Del Pozo-Cruz B., Del Pozo-Cruz J., Sañudo B., Rogers M.E. (2014). Test-retest reliability and minimal detectable change scores for fitness assessment in older adults with type 2 diabetes. Rehabil. Nurs..

[37] Tsai C.L., Chen Z.R., Chia P.S., Pan C.Y., Tseng Y.T., Chen W.C. (2024). Acute resistance exercise combined with whole body vibration and blood flow restriction: Molecular and neurocognitive effects in late-middle-aged and older adults. Exp. Gerontol..

[38] Guadarrama-Molina E., Barrón-Gámez C.E., Estrada-Bellmann I., Meléndez-Flores J.D., Ramírez-Castañeda P., Hernández-Suárez R.M.G., Menchaca-Pérez M., Salas-Fraire O. (2021). Comparison of the effect of whole-body vibration therapy versus conventional therapy on functional balance of patients with Parkinson’s disease: Adding a mixed group. Acta Neurol. Belg..

[39] Lin T.T., Cheng L.Y., Chen C.C., Pan W.R., Tan Y.K., Chen S.F., Wang F.C. (2024). Age-related influence on static and dynamic balance abilities: An inertial measurement unit-based evaluation. Sensors.

[40] Kordi Yoosefinejad A., Shadmehr A., Olyaei G., Talebian S., Bagheri H. (2014). The effectiveness of a single session of Whole-Body Vibration in improving the balance and the strength in type 2 diabetic patients with mild to moderate degree of peripheral neuropathy: A pilot study. J. Bodyw. Mov. Ther..

[41] Mahbub M.H., Hase R., Yamaguchi N., Hiroshige K., Harada N., Bhuiyan A.N.H., Tanabe T. (2020). Acute Effects of Whole-Body Vibration on Peripheral Blood Flow, Vibrotactile Perception and Balance in Older Adults. Int. J. Environ. Res. Public Health.

[42] Xu L., Cardinale M., Rabotti C., Beju B., Mischi M. (2016). Eight-Week Vibration Training of the Elbow Flexors by Force Modulation: Effects on Dynamic and Isometric Strength. J. Strength. Cond. Res..

